# Overview of Metformin and Neurodegeneration: A Comprehensive Review

**DOI:** 10.3390/ph18040486

**Published:** 2025-03-28

**Authors:** Weronika Kruczkowska, Julia Gałęziewska, Paulina Buczek, Elżbieta Płuciennik, Mateusz Kciuk, Agnieszka Śliwińska

**Affiliations:** 1 Department of Functional Genomics, Faculty of Medicine, Medical University of Lodz, Żeligowskiego 7/9, 90-752 Lodz, Poland; weronika.kruczkowska@stud.umed.lodz.pl (W.K.); julia.galeziewska@stud.umed.lodz.pl (J.G.); paulina.buczek@stud.umed.lodz.pl (P.B.); elzbieta.pluciennik@umed.lodz.pl (E.P.); 2 Department of Molecular Biotechnology and Genetics, Faculty of Biology and Environmental Protection, University of Lodz, Banacha Street 12/16, 90-237 Lodz, Poland; mateusz.kciuk@biol.uni.lodz.pl; 3 Department of Nucleic Acid Biochemistry, Medical University of Lodz, Pomorska 251, 92-213 Lodz, Poland

**Keywords:** metformin, neurodegeneration, type 2 diabetes

## Abstract

This comprehensive review examines the therapeutic potential of metformin, a well-established diabetes medication, in treating neurodegenerative disorders. Originally used as a first-line treatment for type 2 diabetes, recent studies have begun investigating metformin’s effects beyond metabolic disorders, particularly its neuroprotective capabilities against conditions like Parkinson’s disease, Alzheimer’s disease, Huntington’s disease, and multiple sclerosis. Key findings demonstrate that metformin’s neuroprotective effects operate through multiple pathways: AMPK activation enhancing cellular energy metabolism and autophagy; upregulation of antioxidant defenses; suppression of inflammation; inhibition of protein aggregation; and improvement of mitochondrial function. These mechanisms collectively address common pathological features in neurodegeneration and neuroinflammation, including oxidative stress, protein accumulation, and mitochondrial dysfunction. Clinical and preclinical evidence supporting metformin’s association with improved cognitive performance, reduced risk of dementia, and modulation of pathological hallmarks of neurodegenerative diseases is critically evaluated. While metformin shows promise as a therapeutic agent, this review emphasizes the need for further investigation to fully understand its mechanisms and optimal therapeutic applications in neurodegenerative diseases.

## 1. Introduction

Metformin, a drug from the biguanide group, has garnered increasing attention for its robust potential in various disease treatments. During the Middle Ages, *Galega officinalis* (goat’s rue) was used for diabetes, which later led to the isolation of guanidine and discovery of its blood sugar-lowering effects in 1918. This ultimately resulted in the synthesis of metformin, published in *Journal of the Chemical Society, Transactions* in 1922, and its successful clinical trials by Jean Sterne in 1957 [1,2,3]. The original use of metformin was as a first-line treatment for type 2 diabetes. In the following years, additional benefits of metformin treatment were observed, such as the prevention of cancer development, reduction in the risk of cardiovascular diseases, life prolongation, and improving fertility in women with polycystic ovary syndrome (PCOS). Recent studies have begun to investigate its effects beyond metabolic disorders, with a focus on its protective potential against neurodegenerative diseases (NDDs) like Parkinson’s disease (PD) and Alzheimer’s disease (AD). Additionally, new research suggests that it may help treat multiple sclerosis by lowering oxidative stress and inflammation in the central nervous system [4,5,6]. Preliminary research suggests that metformin’s ability to modulate mitochondrial function and reduce oxidative stress may offer therapeutic potential in Huntington’s disease (HD), though clinical evidence remains limited. Clinical studies suggest that metformin may improve not only mortality, but also cognitive function and memory in diabetic patients, and is associated with a lower risk of developing dementia [7,8,9,10,11]. It exerts its beneficial effects through a multifaceted mechanism, including AMP-activated protein kinase (AMPK) activation, enhanced antioxidant defenses, and potent anti-inflammatory actions [4,12]. These effects, coupled with its potential to modulate the gut microbiome and promote neurogenesis, suggest a significant impact on cognitive function [13,14]. Clinical and preclinical evidence supports its association with improved cognitive performance, a reduced risk of dementia, and advancement of pathological hallmarks of neurodegenerative diseases. Overall, even though metformin shows promise, more investigation is required to completely understand its processes and therapeutic uses in neurology [15,16]. This review will delve into the multifaceted mechanisms of metformin’s neuroprotective effects and critically evaluate the existing evidence to inform future research directions.

This review article focuses on potential use of metformin in Alzheimer’s disease, Parkinson’s disease, Huntington’s disease, and multiple sclerosis (MS). These conditions collectively encompass key neurodegenerative processes including protein misfolding and aggregation (AD, PD, HD), mitochondrial dysfunction (particularly in PD), oxidative stress (common across all), and neuroinflammation (prominent in MS). Furthermore, these diseases represent different anatomical patterns of neurodegeneration: cortical (AD), subcortical (PD), both cortical and subcortical (HD), and demyelinating (MS). This selection provides a comprehensive framework to evaluate metformin’s multifaceted mechanisms across diverse neurodegenerative pathways, while maintaining a focused analysis of conditions with substantial clinical and preclinical research data.

## 2. Metformin: Basic Pharmacology and Mechanisms

Metformin (N, N-dimethylbiguanide) exhibits complex pharmacokinetics, characterized by slow absorption in the upper small intestine via organic cation transporters (OCT1/3, PMAT) [17,18]. Oral bioavailability ranges from 50 to 60%, with peak plasma concentrations reached within 1–3 h. The drug undergoes minimal metabolism and is primarily eliminated unchanged through renal tubular secretion, with a plasma half-life of 4–9 h [19,20,21]. At therapeutic doses (1500–2500 mg/day), plasma concentrations typically range 1–4 μg/mL. The drug accumulates preferentially in the liver through OCT1-mediated uptake, achieving hepatic concentrations 3–5 times higher than plasma. This tissue distribution pattern aligns with its primary site of action [19,22].

Metformin’s primary effect is mediated through inhibition of hepatic gluconeogenesis [23,24]. While it exhibits effects on cellular metabolism, its primary molecular target has been identified as mitochondrial glycerophosphate dehydrogenase (GPD2), rather than complex I inhibition or AMPK activation which only occur at supra-pharmacological concentrations [25,26,27]. The drug’s action involves modulation of the cellular redox balance, where GPD2 inhibition affects the shuttle of redox equivalents from cytosolic to mitochondrial nicotinamide adenine dinucleotide (NAD) + hydrogen (H) (NADH) [28,29,30]. This leads to a reduced supply of dihydroxyacetone phosphate from glycerol and lower adenosine triphosphate (ATP) levels due to a decreased redox equivalent transfer into the mitochondrial matrix [25,28,31]. Additionally, the elevated NADH/NAD+ ratio in the cytosol inhibits lactate dehydrogenase, reducing the conversion of lactate to the gluconeogenic precursor pyruvate [25,32]. While this mechanism effectively reduces hepatic glucose production, it may also impact lactate metabolism, potentially leading to lactate acidosis [33,34]. The drug’s effects on other hepatic metabolic functions, particularly regarding hepatic steatosis, remain under investigation with controversial results [35].

### 2.1. Chemical Structure and Properties

**Figure 1:**
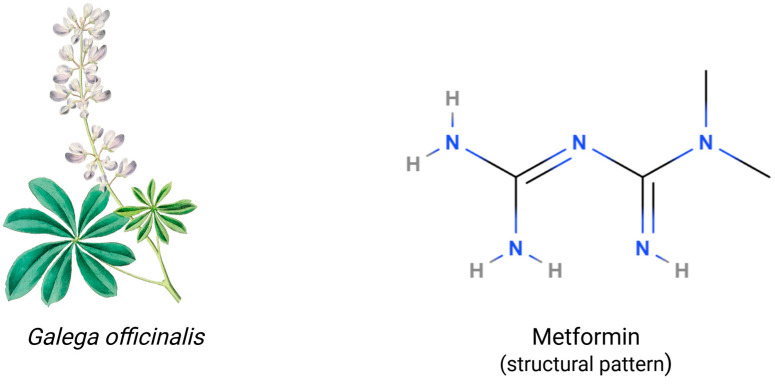
Structural and botanical characterization of metformin (C_4_H_11_N_5_). *Galega officinalis* (Fabaceae), the plant from which metformin was originally derived, is characterized by palmately compound leaves and racemose inflorescences. The molecular structure of metformin shows a structural pattern that highlights the biguanide scaffold with terminal N-methylation. Image by rawpixel.com on Freepik. Created in BioRender. Kciuk, M. (2025) https://BioRender.com/o18q303 (accessed on 9 March 2025).

The chemical structure of metformin contains two linked guanidine groups forming a biguanide scaffold, whose planar structure enables specific molecular interactions, e.g., hydrogen bonding capacity and charge distribution [25,36]. Moreover, the characteristic formation of metformin molecules acquires resonance stabilization across N-C bonds and delocalizes positive charge distribution [37,38]. The structure of metformin is illustrated in Figure 1. Metformin’s effectiveness is primarily driven by its hydrophilic nature and positive charge at physiological pH, which enables efficient transport through organic cation transporters and cellular membranes. Its small molecular size (129.16 g/mol) and planar biguanide structure, combined with high chemical stability and minimal protein binding, allow for predictable pharmacokinetics and sustained therapeutic action [19,39]. Metformin exhibits pH-dependent solution behavior, existing primarily as a cation at physiological pH with multiple possible protonation states that critically affect its tissue distribution and interaction with membrane transporters [40,41,42]. The compound exhibits good photostability and a defined thermal stability range, but is sensitive to humidity. Therefore, specific storage conditions are required, including protection from heat and moisture, controlled relative humidity, and appropriate packaging to maintain potency [19,37,43]. These physicochemical properties fundamentally determine metformin’s pharmaceutical performance and therapeutic efficacy.

Metformin’s ability to cross the blood–brain barrier (BBB) is relatively limited, with only 10–20% of the drug reaching the central nervous system [44]. Levodopa, a known drug used in Parkinson’s disease, has effective penetration, but when administered alone its peripheral decarboxylation to dopamine by aromatic amino acid decarboxylase is rapid, reducing the amount of levodopa available for BBB transport. However, levodopa crosses the BBB effectively when combined with peripheral decarboxylase inhibitors like carbidopa or benserazide, which prevent its premature metabolism [45]. In contrast, anti-epileptic drugs (AEDs) like phenytoin and carbamazepine achieve significantly higher penetration, approximately 50–60% and 40–50%, respectively, due to their lipophilic properties. Valproic acid, another AED, shows moderate BBB penetration (similar to metformin and levodopa), at 10–20%. This highlights the variability in CNS drug delivery, with lipophilicity and specific transporter interactions playing crucial roles [46,47]. Metformin’s restricted penetration is primarily due to its hydrophilic nature and reliance on specific transport mechanisms. The drug primarily depends on organic cation transporters (OCTs), particularly OCT1 and OCT3, as well as the plasma membrane monoamine transporter (PMAT) to cross the BBB. However, these transporters have relatively low expression at the BBB compared to their abundance in peripheral tissues, such as the liver and kidneys [25,48]. Additionally, P-glycoprotein (P-gp) efflux transporters actively pump metformin back into the bloodstream, further limiting its brain accumulation [49,50]. Levodopa, utilizing the large neutral amino acids transporter small subunit 1 (LAT1) transporter, efficiently crosses the BBB, contrasting with metformin’s limited permeability via organic cation transporters [51,52]. Anti-epileptic drugs such as gabapentin can leverage specific transporters like LAT1, while others may be hindered by P-glycoprotein (P-gp)-mediated efflux. Conversely, lipophilic small molecules often achieve a wide CNS distribution through passive diffusion, circumventing the need for specific transporters. These observations underscore the importance of considering drug physicochemical properties and transporter affinities in the design and delivery of effective CNS therapeutics [53,54,55].

Metformin shows potential for treating neurodegenerative diseases, but its hydrophilic nature and limited blood–brain barrier penetration necessitate prolonged exposure and repeated dosing. Despite this, it has a well-established safety profile from decades of clinical use in T2DM. However, its long-term safety, particularly in patients with or without metabolic syndrome comorbidities, requires careful evaluation [56,57,58].

Metformin is generally well-tolerated, with transient gastrointestinal side effects like nausea, diarrhea, and abdominal discomfort, which can be managed through dose titration or extended-release formulations. It does not cause hypoglycemia when used alone, making it safer than many other antidiabetic drugs [59,60,61]. However, rare but serious complications like metformin-associated lactic acidosis (MALA) must be considered. MALA is extremely rare, occurring in 3–10 cases per 100,000 patient-years, primarily in those with renal impairment, hepatic dysfunction, or hypoxia-related conditions. Regular renal function monitoring is recommended for long-term use [34,62].

Another concern is vitamin B12 deficiency due to impaired absorption, which can lead to peripheral neuropathy or cognitive impairment, especially in those at risk of NDDs. Chronic metformin users have lower B12 levels than non-users, with deficiency rates of 5–10%. Regular monitoring and supplementation can help mitigate this risk [63,64].

Higher doses or longer treatment durations may be needed to achieve therapeutic CNS concentrations for NDDs like Alzheimer’s and Parkinson’s, raising concerns about systemic toxicity, particularly in elderly populations. While metformin is generally safe for long-term use in diabetics, its safety in non-diabetics for neuroprotection requires further study [65,66]. Emerging drug delivery strategies, such as nanocarrier-based formulations or intranasal delivery, aim to enhance BBB penetration while minimizing systemic exposure. These approaches could optimize therapeutic efficacy while reducing the risks associated with high-dose regimens [67,68].

Despite this limited BBB penetration, metformin still exhibits significant neuroprotective effects through both direct central nervous system actions (via the fraction that does cross the BBB) and indirect peripheral mechanisms that influence brain function through systemic metabolic and inflammatory pathways [69,70]. To overcome these limitations, current research focuses on developing modified formulations with enhanced BBB penetration, including lipophilic derivatives, nanocarrier delivery systems, and alternative administration routes such as intranasal delivery [71,72,73].

### 2.2. Traditional Use in Diabetes

The biguanide metformin represents the established first-line pharmacological intervention for type 2 diabetes mellitus (T2DM), with extensive clinical validation spanning several decades [74,75]. Clinical efficacy manifests primarily through significant reductions in glycated hemoglobin (HbA1c), typically achieving 1.0–2.0 percentage point decrements in controlled studies [76]. This therapeutic agent demonstrates particular utility in treatment-naive patients, especially those presenting with obesity or metabolic syndrome comorbidities [77]. The compound’s clinical profile is distinguished by several advantageous characteristics, e.g., neutral or favorable effects on body mass index, minimal risk of hypoglycemic events, and robust tolerability in most patient populations [78,79,80]. The medication’s extensive clinical history and favorable benefit–risk profile have established it as the cornerstone of T2DM pharmacological management. Recent investigations have suggested potential pleiotropic benefits beyond glycemic control [81,82].

### 2.3. Key Molecular Mechanisms

The molecular basis for metformin’s therapeutic effects centers on multiple interconnected pathways. While AMP-activated protein kinase activation serves as an important mediator, metformin also exerts significant AMPK-independent effects [42,83]. This complex network of molecular actions initiates a cascade of cellular responses that collectively improve glucose homeostasis [84]. The key mechanisms of metformin action include the suppression of hepatic glucose production, enhancement of peripheral insulin sensitivity, modulation of intestinal glucose metabolism, and regulation of cellular energy metabolism, as is summarized in Table 1.

These mechanisms have therapeutic implications, especially for metformin timing and dosing. While metformin shows limited blood–brain barrier penetration, its beneficial effects on neurodegeneration appear to work through both direct and indirect pathways [44,104]. The indirect effects stem from its action on peripheral tissues—particularly liver and gut—where it reduces systemic inflammation, improves insulin sensitivity, and decreases oxidative stress, all of which positively impact brain health [13,105]. Additionally, metformin directly alleviates endoplasmic reticulum (ER) stress in neurons by activating the AMPK pathway and enhancing the unfolded protein response (UPR), which is particularly significant for neurodegenerative conditions where protein misfolding plays a central role [106,107,108]. These molecular mechanisms, along with metformin’s anti-inflammatory properties (which are particularly relevant in neurodegeneration), operate synergistically to improve glycemic control while simultaneously affecting multiple metabolic pathways involved in energy homeostasis [56,109]. The complex interplay between these mechanisms contributes to metformin’s broad therapeutic efficacy and emerging pleiotropic effects, including reduced cancer risk, improved cardiovascular outcomes, increased longevity, and neuroprotective properties [25,110,111,112].

Recent metformin research has significantly expanded our understanding of its mechanisms and therapeutic potential. Beyond traditional AMPK activation, studies reveal metformin’s influence on lysosomal pathways, redox states, and its interaction with specific genetic variants that impact individual responses. This has led to the identification of novel molecular targets and a deeper understanding of its glucose-lowering effects [113]. Metformin’s impact extends to modulating the gut microbiota, influencing bile acid metabolism, and affecting incretin secretion, further contributing to its multifaceted glucose-lowering effects. Specifically, the article reinforces metformin’s role in altering gut microbial composition, which in turn impacts glucose homeostasis. Furthermore, investigations into metformin’s benefits beyond diabetes, including cardiovascular protection and anti-inflammatory properties, are broadening its clinical applications. Ongoing research focuses on leveraging genetic profiling for personalized metformin therapy, exploring new drug targets based on its diverse actions, and even considering innovative approaches like mitochondrial transfer therapies to combat diabetic mitochondrial dysfunction [25,114,115].

## 3. Molecular Mechanism of Metformin Action in the Liver

There is a general consensus that the glucose-lowering impact in T2DM patients is primarily mediated by the inhibition of hepatic gluconeogenesis. However, after decades of research, the mechanism by which metformin suppresses this process remains contentious. Widely studied mechanisms of metformin action, such as complex I inhibition (reviewed by Fontaine in 2018) leading to 5′AMPK activation, have only been observed at supra-pharmacological (>1 mM) metformin concentrations, which do not occur in the clinical setting [23,116]. Furthermore, animal models imply that AMPK is not required for metformin’s anti-hyperglycemic actions, as mice with liver-specific genetic deletions of AMPK or the upstream kinase ‘liver kinase B1’ can still reduce hepatic gluconeogenesis in response to metformin [106,117,118].

Based on the discovery that metformin changes the cellular redox equilibrium, various groups have described a redox-dependent mode of action: Metformin inhibits the enzyme mitochondrial glycerophosphate dehydrogenase (GPD2), which, along with cytosolic glycerophosphate dehydrogenase (GPD1), catalyzes the transfer of redox equivalents from cytosolic to mitochondrial NADH during the reduction of glycerophosphate to dihydroxyacetone phosphate, an intermediate in the gluconeogenetic pathway. Reduced supply of dihydroxyacetone phosphate from glycerol, as well as a decrease in ATP levels caused by reduced transfer of redox equivalents into the mitochondrial matrix, limit gluconeogenesis [25,26,28].

Furthermore, an increased NADH/NAD+ cytosolic ratio inhibits the enzyme lactate dehydrogenase, which converts lactate to the gluconeogenetic precursor pyruvate. A possible negative effect of this method of action is impaired lactate recycling to glucose (Cori cycle), which increases the risk of lactic acidosis [33,119]. It is unknown whether metformin can have a significant temporary effect on other metabolic activities of the liver. In vitro and in vivo studies on metformin’s capacity to alleviate hepatic steatosis have yielded conflicting results [35].

## 4. Neurodegenerative Diseases: Common Pathological Mechanisms

While distinct in their primary manifestations, neurodegenerative conditions such as Alzheimer’s disease, Parkinson’s disease, and Huntington’s disease share several key pathological mechanisms. Multiple sclerosis, while primarily an autoimmune inflammatory condition of the central nervous system, shares several of these same pathological mechanisms that ultimately contribute to neurodegeneration. Despite their different etiologies, all four conditions demonstrate overlapping features such as oxidative stress, mitochondrial dysfunction, neuroinflammation, and eventual neuronal loss [120,121,122].

Activated microglia and astrocytes release inflammatory mediators that can harm neurons and myelin in all four illnesses, a process known as neuroinflammation. There are key functions for protein misfolding and aggregation; amyloid beta and tau in Alzheimer’s disease, α-synuclein in Parkinson’s disease, mutant huntingtin in Huntington’s disease, and maybe misfolded proteins that contribute to autoimmune reactions in multiple sclerosis [123,124]. All of these disorders are characterized by oxidative stress and mitochondrial malfunction, which result in energy deficiencies and the generation of toxic reactive oxygen species that destroy cellular components [125,126]. Axonal transport disruption and synaptic dysfunction are also features of each illness, although they are caused by diverse molecular mechanisms: tau in Alzheimer’s, α-synuclein in Parkinson’s, huntingtin in Huntington’s, and inflammation in multiple sclerosis [127,128].

Each disease is characterized by the death of particular neuronal populations, such as oligodendrocytes with subsequent neuronal damage in multiple sclerosis, dopaminergic neurons in Parkinson’s, striatal and cortical neurons in Huntington’s, and hippocampus and cortical neurons in Alzheimer’s [129,130]. Lastly, all of these disorders exhibit signs of disturbed proteostasis and compromised cellular quality control systems, such as autophagy pathways and the ubiquitin–proteasome system [131].

### 4.1. Protein Aggregation

In neurodegenerative illnesses, protein aggregation is a key pathogenic process. These illnesses are typified by the build-up of misfolded proteins that congregate and cause neurodegeneration and cellular malfunction [132].

Usually, this process starts when proteins change from their original structural configuration for a variety of reasons, including oxidative stress, post-translational changes, or genetic abnormalities that impact protein homeostasis. The aggregation pathway typically occurs in phases: first, hydrophobic areas that are ordinarily hidden inside the protein’s tertiary structure are exposed by first protein misfolding, resulting in the production of oligomeric species that act as nucleation sites for additional aggregation [133,134,135]. After forming more ordered structures like protofibrils, these oligomers can develop into amyloid fibrils, which are characterized by their abundance of β-sheets. These protein aggregates may have prion-like characteristics, spreading sickness to nearby cells via methods of cell-to-cell transmission and accelerating the course of the illness. “Seeding” describes how misfolded protein “templates” spread between cells, converting normal proteins into the misfolded state and triggering a chain reaction of aggregation [136,137,138]. This spread occurs via exosomes, tunneling nanotubes, and synaptic transmission. The resulting aggregates disrupt key cellular processes—protein quality control, mitochondrial function, axonal transport, and synaptic transmission—ultimately causing neuronal dysfunction and death [139].

Notably, different neurodegenerative conditions exhibit disease-specific protein aggregates: α-synuclein in Parkinson’s disease forms Lewy bodies, amyloid beta and tau in Alzheimer’s disease produce extracellular plaques and intracellular neurofibrillary tangles, respectively, while the huntingtin protein with expanded polyglutamine repeats aggregates in Huntington’s disease. While the exact misfolded protein in multiple sclerosis (MS) is not yet identified, research indicates that protein misfolding might contribute to the disease’s development. Studies have found soluble oligomers in the brains and cerebrospinal fluid of MS patients, similar to those seen in Alzheimer’s disease [140,141,142,143,144].

Protein aggregation is caused by intricate and varied molecular processes that involve both environmental and cellular influences [145]. Under normal circumstances, cellular quality control systems—such as the autophagy–lysosomal pathway, the ubiquitin–proteasome system, molecular chaperones, and the endoplasmic reticulum stress response system, including the unfolded protein response—are essential in preventing protein aggregation and maintaining proteostasis [146,147]. However, when people age or experience sickness, these defenses may be overtaxed or stop working, leading to the accumulation of misfolded proteins in the ER lumen, which triggers ER stress, activates stress sensors (IRE1α, PERK, and ATF6), and initiates the UPR signaling cascade. If this stress persists and cannot be resolved, it can lead to chronic ER stress, further compromising protein homeostasis and potentially triggering cell death pathways [148,149]. Phosphorylation, ubiquitination, and acetylation are examples of post-translational changes that can have a major impact on the toxicity and propensity of proteins to aggregate. Moreover, oxidative stress induction and direct protein binding are two of the ways that metal ions—in particular, copper, zinc, and iron—have been linked to speeding up protein aggregation [150,151,152,153].

**Figure 2:**
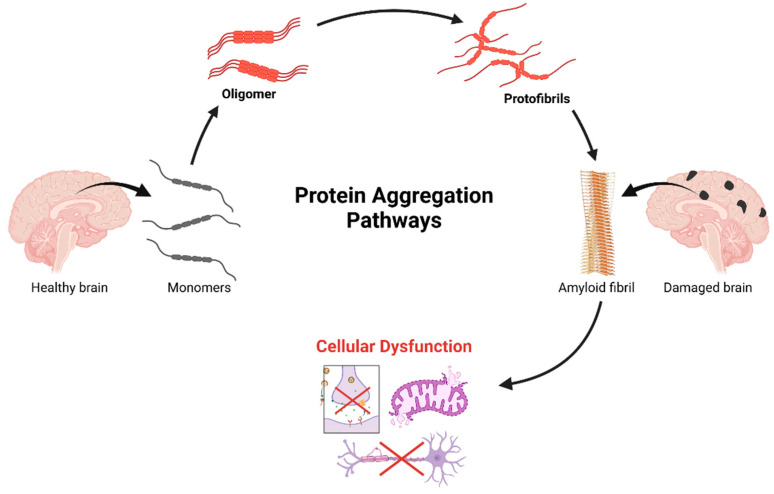
A cascade of progressive protein aggregation in neurodegenerative pathogenesis. The process initiates in the healthy brain with native protein monomers, which undergo conformational changes leading to oligomerization. These oligomeric intermediates further aggregate into protofibrils, ultimately assembling into highly ordered amyloid fibrils. This terminal aggregation state correlates with observable neuropathology, characterized by distinct patterns of brain damage. The downstream of cellular consequences of protein aggregation, includes disruption of essential neuronal functions: protein quality control mechanisms, mitochondrial homeostasis, axonal transport efficiency, and synaptic transmission integrity. This cascade represents a crucial mechanistic framework in understanding the molecular basis of neurodegenerative disorders and identifies potential therapeutic intervention points along the aggregation pathway. Created in BioRender. Kciuk, M. (2025) https://BioRender.com/g29m614 (accessed on 19 February 2025).

Protein aggregate toxicity arises from multiple mechanisms, which is illustrated in Figure 2. Oligomers, not fibrils, are the main culprits, disrupting cell membranes, calcium balance, and triggering inflammation. They also sequester essential proteins (e.g., transcription factors, chaperones), impairing cellular function [154,155,156]. Aggregates activate stress responses, causing chronic stress and cell death. Their accumulation fuels neuroinflammation via microglial and astrocyte activation, worsening neurodegeneration [157,158,159,160].

### 4.2. Oxidative Stress

Oxidative stress (OS) occurs when there is an imbalance between the production of reactive oxygen species (ROS) and the body’s ability to neutralize them with antioxidants. ROS, which include free radicals like superoxide anion (O_2_^−^), hydroxyl radical (OH·), and non-free radicals such as hydrogen peroxide (H_2_O_2_), are produced during normal cellular metabolism, particularly in the mitochondria through the electron transport chain [161,162]. Under physiological conditions, ROS play crucial roles in cell signaling and homeostasis; however, excessive ROS can lead to cellular damage by oxidizing proteins, lipids, and nucleic acids [125,163].

Sources of ROS include mitochondrial respiration, inflammation, and exogenous factors like pollution and radiation, but interestingly, hyperglycemia as well [164,165]. In neurodegenerative conditions, mitochondrial dysfunction and neuroinflammation lead to increased ROS generation. These reactive species damage cellular components, including proteins, lipids, and DNA. For example, in Alzheimer’s disease, amyloid beta oligomers induce oxidative stress, leading to lipid peroxidation and neuronal membrane damage. Furthermore, ROS activate glial cells, triggering the release of pro-inflammatory cytokines that exacerbate neuronal injury. This creates a vicious cycle where inflammation amplifies oxidative stress, further promoting neuronal damage. Protein misfolding and aggregation, as seen in Parkinson’s disease with alpha-synuclein, are also exacerbated by oxidative modifications. The accumulation of oxidized DNA bases further impairs neuronal function and survival. Understanding these intricate mechanisms provides crucial insights for developing therapeutic strategies aimed at enhancing antioxidant defenses and targeting specific pathways involved in ROS-mediated neuronal damage, with the goal of mitigating disease progression [166,167].

### 4.3. Neuroinflammation

Neuroinflammation, a complex immune response in the central nervous system, is crucial in the development and progression of neurodegenerative diseases. It involves the activation of microglia and astrocytes, which undergo significant changes in form and function. Microglia, the brain’s immune cells, shift from a resting state to an active state, increasing their ability to engulf debris, producing pro-inflammatory molecules, and altering their gene expression. This activation is triggered by factors like misfolded proteins, cellular debris, and damage-associated molecular patterns (DAMPs), all linked to neurodegeneration [168,169,170,171].

Similarly, astrocytes experience reactive astrogliosis, which is characterized by pro- and anti-inflammatory factor production, cellular hypertrophy, and elevation of glial fibrillary acidic protein (GFAP) [172]. In the setting of neurodegeneration, the astrocytic response involves the creation of a glial scar, which can have both beneficial and harmful effects. Reactive oxygen species, chemokines, nitric oxide, pro-inflammatory cytokines (IL-1β, TNF-α, and IL-6), and other inflammatory mediators are released by these activated glial cells, resulting in a neuroinflammatory milieu that can have a major effect on neuronal survival and function [173,174].

An important factor in neuroinflammation is the blood–brain barrier, whose disruption frequently contributes to and results from inflammatory processes. Peripheral immune cells such as T lymphocytes, monocytes, and neutrophils can infiltrate when the BBB is compromised, which exacerbates the inflammatory response [157,175]. Through intricate signaling networks, these invading cells engage with native CNS cells, intensifying the inflammatory cascade and possibly worsening tissue damage. In addition to causing the extravasation of plasma proteins and the activation of complement cascades, the compromise of BBB integrity also contributes to the inflammatory environment [176].

**Figure 3:**
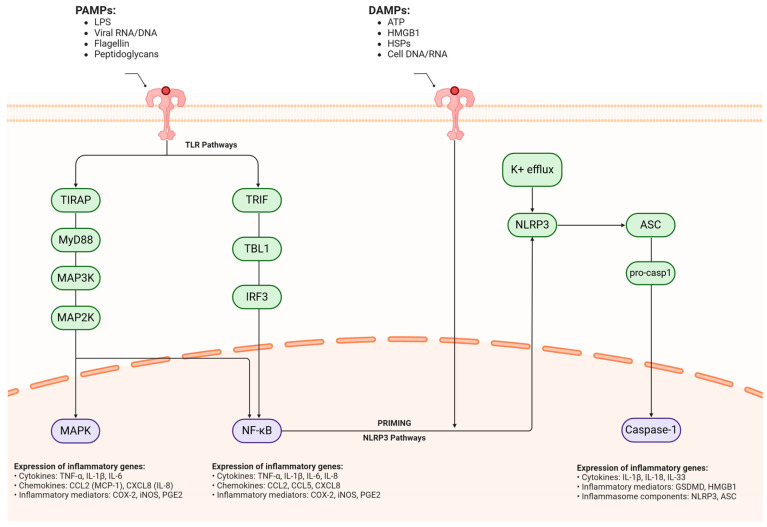
Molecular mechanisms of neuroinflammatory signaling cascades in neurodegeneration. Converging molecular pathways mediating neuroinflammatory responses in neurodegenerative conditions have two primary inflammatory triggers: pathogen-associated molecular patterns (PAMPs) and damage-associated molecular patterns (DAMPs), which initiate distinct but interconnected signaling cascades across the cellular membrane. PAMPs (pathogen-associated molecular patterns), including LPS, viral RNA/DNA, flagellin, and peptidoglycans, initiate inflammation through TLR-mediated signaling. This process branches into two main cascades: TIRAP–MyD88-MAP3K–MAP2K–MAPK and TRIF–TBL1–IRF3. Both pathways converge to activate NF-κB, triggering the expression of pro-inflammatory genes. These include critical cytokines (TNF-α, IL-1β, IL-6, IL-8), chemokines (CCL2, CXCL8), and inflammatory mediators (COX-2, iNOS, PGE2). Simultaneously, DAMPs (damage-associated molecular patterns) such as ATP, HMGB1, HSPs, and cellular DNA/RNA activate a separate inflammatory cascade through the NLRP3 inflammasome. This process begins with potassium efflux, which triggers NLRP3 activation, followed by ASC recruitment and pro-caspase-1 processing. The end result is active caspase-1, which promotes the expression of inflammatory mediators, particularly GSDMD and HMGB1. PAMPs = pathogen-associated molecular patterns, LPS = lipopolysaccharide, RNA = ribonucleic Acid, DNA = deoxyribonucleic acid, TLR = toll-like receptor, TIRAP = TIR domain-containing adaptor protein, MyD88 = myeloid differentiation primary response 88, MAP3K = mitogen-activated protein kinase kinase kinase, MAP2K = mitogen-activated protein kinase kinase, MAPK = mitogen-activated protein kinase, TRIF = TIR domain-containing adapter-inducing interferon-β, TBL1 = transducin beta-like 1, IRF3 = interferon regulatory factor 3, NF-κB = nuclear factor kappa-light-chain-enhancer of activated B cells, TNF-α = tumor necrosis factor alpha, IL-1β = interleukin-1 beta, IL-6 = interleukin-6, IL-8 = interleukin-8, CCL2 = C-C motif chemokine ligand 2, CXCL8 = C-X-C motif chemokine ligand 8, COX-2 = cyclooxygenase-2, iNOS = inducible nitric oxide synthase, PGE2 = prostaglandin E2, DAMPs = damage-associated molecular patterns, ATP = adenosine triphosphate, HMGB1 = high mobility group box 1, HSPs = heat shock proteins, NLRP3 = NOD-like receptor protein 3, ASC = apoptosis-associated speck-like protein containing a CARD, GSDMD = gasdermin D, K+ = potassium. Created in BioRender. Kciuk, M. (2025) https://BioRender.com/zb9c38p (accessed on 27 March 2025).

Neuroinflammation features complex molecular pathways. Pattern recognition receptors (PRRs), such as Toll-like receptors (TLRs) and NOD-like receptors (NLRs), detect pathogen-associated molecular patterns (PAMPs), triggering the activation of inflammatory transcription factors like NF-κB and AP-1. This, in turn, leads to the increased expression of pro-inflammatory genes. Inflammasome complexes, particularly NLRP3, play a critical role in processing and releasing mature IL-1β and IL-18, further propagating the inflammatory cascade [177,178,179]. Chronic neuroinflammation establishes a self-sustaining cycle of cellular damage and immune activation. The persistent activation of glial cells, including microglia and astrocytes, results in the sustained production of neurotoxic factors, such as pro-inflammatory cytokines, reactive oxygen species, and excitotoxic molecules like glutamate. This chronic inflammatory state can significantly impair synaptic function, disrupt neuronal networks, and ultimately contribute to neurodegeneration [180,181,182]. Furthermore, the inflammatory environment can influence protein aggregation and clearance mechanisms, potentially exacerbating the accumulation of pathogenic protein species characteristic of various neurodegenerative disorders, such as amyloid beta in Alzheimer’s disease or alpha-synuclein in Parkinson’s disease. This interplay between inflammation and protein aggregation creates a vicious cycle that fuels disease progression [177,183]. This process is depicted in Figure 3.

### 4.4. Mitochondrial Dysfunction

Given the high energy requirements of neurons and their dependence on oxidative phosphorylation for ATP synthesis, mitochondrial dysfunction is a key pathogenic factor in neurodegenerative diseases. In addition to their canonical function in bioenergetics, these organelles are key modulators of apoptotic pathways, redox signaling, and calcium homeostasis inside cells. Numerous interrelated pathways contribute to the reduced function of mitochondria in neurodegenerative diseases, resulting in a complex network of cellular disturbances that ultimately lead to neuronal death [184,185].

One of the main signs of mitochondrial impairment is the malfunction of the electron transport chain (ETC), which is typified by decreased activity of respiratory chain complexes, especially Complex I and IV. Reduced ATP synthesis and increased electron leakage are the results of this malfunction, which raises the creation of reactive oxygen species (ROS). A vicious loop is created by the increased generation of ROS, which can exacerbate the initial malfunction by further damaging proteins, lipids, and mitochondrial DNA (mtDNA). Because of its close proximity to the ETC and its lack of adequate repair mechanisms, mtDNA is especially susceptible to oxidative damage, which can result in accumulating mutations that further impair respiratory chain function [186,187,188].

Mitochondrial quality control is essential for neuronal health and becomes disrupted in neurodegenerative diseases. The balance between mitochondrial fusion and fission proteins becomes impaired, affecting mitochondrial morphology and function [189,190]. This disruption compromises mitophagy, the process that removes damaged mitochondria, as demonstrated by PINK1 and Parkin mutations in Parkinson’s disease. Additionally, mitochondrial calcium regulation becomes dysfunctional, leading to dangerous cytoplasmic calcium accumulation that triggers excitotoxicity and cell death pathways, particularly through the mitochondrial permeability transition pore [191,192].

Mitochondrial transport along neurons, crucial for synaptic function, is also compromised in these diseases. This disruption, affecting proteins like kinesin and dynein, causes energy deficits at synapses and contributes to synapse loss. Disease-specific protein aggregates like α-synuclein and amyloid beta can directly impair mitochondrial function, creating a destructive feedback loop [193,194]. These insights have led to therapeutic strategies including antioxidants, biogenesis enhancers, and modulators of mitochondrial dynamics. Early intervention and reliable biomarkers are crucial for treatment success. However, developing effective therapies remains challenging due to the complexity of mitochondrial dysfunction and diverse neuronal needs [195].

### 4.5. Insulin Resistance in the Brain

An increasingly acknowledged pathogenic mechanism causing neurodegenerative diseases, including Alzheimer’s disease, is insulin resistance in the brain. As a result, some researchers have proposed the moniker “type 3 diabetes”. Despite having enough insulin present, brain insulin resistance is defined by a decreased response to insulin signaling in neural tissues. This impairs cellular metabolism, synaptic plasticity, and cognitive function. Complex connections between cellular stress responses, inflammatory processes, and metabolic dysfunction underlie the molecular causes of central insulin resistance [5,196,197].

Brain insulin signaling, crucial for neuronal function, involves activating insulin receptors (IR) and IGF-1R, leading to IRS protein phosphorylation and downstream activation of PI3K/Akt and MAPK pathways, affecting glucose metabolism, synaptic plasticity, and cell survival. In insulin resistance, this cascade is compromised due to increased serine/threonine phosphorylation of IRS proteins, reduced tyrosine phosphorylation, and decreased pathway activation [198,199]. Several interconnected processes underlie brain insulin resistance. Inflammatory mediators (TNF-α, IL-6) interfere with signaling via JNK and IKK-β, promoting inhibitory IRS phosphorylation. Oxidative stress, often elevated in neurodegeneration, damages signaling components and creates AGEs, further impairing insulin sensitivity. Lipotoxicity, from aberrant lipid metabolism and ceramide/diacylglycerol accumulation, activates protein kinase C isoforms that disrupt insulin signaling [200,201].

Insulin resistance in the brain has wide-ranging and complex effects. Energy metabolism is compromised by reduced glucose transporter 4 (GLUT4) translocation and decreased glucose uptake in brain cells caused by impaired insulin signaling. Cellular repair processes, synaptic transmission, and neurotransmitter synthesis can all be impacted by this metabolic abnormality [202,203]. Additionally, by decreasing the activation of insulin-dependent protein phosphatases and increasing the activity of glycogen synthase kinase 3β (GSK-3β), insulin resistance can hinder the clearance of amyloid beta and promote tau hyperphosphorylation, directly connecting insulin resistance to the pathological hallmarks of Alzheimer’s disease [204,205].

Peripheral and central insulin resistance share a bidirectional relationship. Systemic metabolic disorders like obesity and type 2 diabetes promote brain insulin resistance through inflammation, oxidative stress, and disrupted adipokine signaling. The blood–brain barrier plays a crucial role, as metabolic stress compromises its function, affecting insulin transport and increasing inflammatory mediator infiltration. Brain insulin resistance impairs synaptic plasticity and cognitive function by disrupting NMDA receptors, AMPA receptor trafficking, and neurotrophic signaling, while also affecting key neurotransmitter systems [206,207].

Various therapeutic approaches have emerged, from insulin sensitizers to intranasal insulin delivery, while lifestyle changes may improve brain insulin signaling. Developing biomarkers for monitoring and understanding the complex interactions with other neurodegenerative mechanisms remains crucial for creating effective treatments [208] 39317178.

### 4.6. Diabetes Mellitus and Neurodegeneration: Shared Pathophysiological Mechanisms

The connection between diabetes mellitus and neurodegenerative diseases highlights shared pathophysiological mechanisms, explaining why antidiabetic drugs like metformin hold therapeutic promise for various neurodegenerative disorders [209,210]. It is also crucial to note that Alzheimer’s disease has been referred to as type 3 diabetes, which stems from shared molecular mechanisms and some risk factors. Chronic hyperglycemia in diabetes triggers neurotoxic processes such as oxidative stress, advanced glycation end product formation, and inflammation, which closely resemble pathological mechanisms in neurodegeneration [211,212]. These metabolic disruptions harm cellular components, accelerate protein misfolding and aggregation, and create a neurochemical environment favorable for neurodegeneration, as evidenced by the significantly higher risk of Alzheimer’s and Parkinson’s disease in diabetic patients. Central insulin resistance further strengthens the link to neurodegenerative pathology by impairing brain insulin signaling, which is crucial for glucose metabolism, synaptic plasticity, neuronal survival, and cognitive function [213,214,215]. The inflammatory interplay between these disorders is especially crucial, as systemic inflammation in diabetes activates microglia, fostering a neuroinflammatory environment similar to that seen in multiple neurodegenerative diseases [216,217]. Another key link is vascular pathology, where diabetic microangiopathy disrupts cerebral circulation, weakens the blood–brain barrier integrity, and accelerates neurodegenerative progression [4,218]. Clinically, these overlapping mechanisms result in faster cognitive decline and an earlier onset of neurodegenerative symptoms in diabetic individuals [219,220]. This biological convergence provides a mechanistic rationale for metformin’s potential to mitigate neurodegenerative processes by targeting multiple shared pathological pathways, paving the way for repurposing this well-established antidiabetic drug for neurodegenerative conditions.

## 5. Molecular Mechanisms of Metformin in Neuroprotection

### 5.1. Metformin in Specific Neurodegenerative Conditions

Metformin has emerged as a promising therapeutic agent in the field of neurodegenerative disorders [56]. Originally developed to control blood glucose levels, this biguanide drug has revealed unexpected neuroprotective properties that extend far beyond its traditional metabolic effects [221].

In recent years, researchers have uncovered compelling evidence suggesting that metformin’s mechanisms of action may offer significant benefits in various neurodegenerative conditions, sparking intense interest in its potential repurposing for brain health [222,223]. The intersection of metabolic regulation and neurodegeneration has become increasingly apparent as scientists uncover the complex relationships between cellular energy metabolism, inflammation, and neuronal health [224]. Metformin’s ability to modulate these fundamental biological processes, particularly through its activation of AMPK signaling pathways and regulation of mitochondrial function as we mentioned before, positions the drug as a valuable candidate for addressing the multifaceted nature of neurodegenerative diseases [84,225,226].

The neuroprotective effects of metformin can be attributed to both common pathogenic pathway-dependent mechanisms and disease-specific modes of action. Metformin exerts significant effects on protein homeostasis by inhibiting protein misfolding and aggregation. Studies have demonstrated that metformin reduces the formation of toxic protein aggregates including α-synuclein, amyloid-β, tau, and huntingtin [227,228]. Furthermore, metformin disrupts the prion-like spreading of misfolded proteins between neurons in mouse models [229]. Metformin also enhances autophagic and proteasomal degradation of misfolded proteins [230]. Neuroinflammation modulation constitutes a significant aspect of metformin’s neuroprotective profile. Metformin inhibits microglial activation and proliferation while reducing pro-inflammatory cytokine production, including TNF-α, IL-1β, and IL-6 [231]. Additionally, studies have found that metformin promotes anti-inflammatory phenotypes in glial cells and suppresses the NF-κB signaling pathway [231,232].

From Alzheimer’s and Parkinson’s disease to multiple sclerosis and Huntington’s disease, research indicates that metformin may influence key pathological processes common to these conditions [112,233,234]. Understanding metformin’s role in neurodegenerative disorders not only offers hope for new therapeutic strategies but also provides valuable insights into the underlying mechanisms of these devastating conditions [224,235]. As the global burden of neurodegenerative diseases continues to grow with an aging population, the exploration of established medications like metformin represents an efficient approach to drug development, potentially accelerating the path to effective treatments for patients in need [236,237].

### 5.2. Alzheimer’s Disease

Metformin has gained interest for its potential effects on Alzheimer’s disease (AD). It activates the AMPK, reducing amyloid beta production and inhibiting neuroinflammation. It also reduces oxidative stress by stabilizing the nuclear factor erythroid 2-related factor 2 (Nrf2) pathway and enhancing antioxidant enzyme expression. Metformin also decreases neuronal apoptosis and improves mitochondrial function, potentially alleviating memory loss associated with AD. However, findings are inconclusive [220,238].

Based on the research literature, T2DM significantly increases the risk of neurodegenerative conditions, particularly Alzheimer’s disease (AD). Interestingly, while T2DM shows these strong connections to neurodegenerative diseases, recent Mendelian randomization studies suggest that type 1 diabetes mellitus (T1DM) may have a different relationship, showing reduced genetic susceptibility to Parkinson’s disease and no significant genetic connection to AD [239,240]. Studies have shown that individuals with T2DM experience a 45% faster decline in cognitive abilities, memory, and reasoning compared to non-diabetic individuals [241,242]. The connection between these conditions is driven by several shared risk factors. These include obesity (which promotes insulin resistance and neuroinflammation), hypertension (which damages blood vessels and impairs brain blood flow), oxidative stress (which affects both glucose metabolism and neuronal function), poor diet (particularly high-fat, high-sugar diets that contribute to insulin resistance and brain inflammation), and a sedentary lifestyle (which reduces insulin sensitivity and is associated with increased amyloid beta deposition). Genetics also plays a role, with certain genes affecting both conditions through shared pathways. Additionally, lifestyle factors such as smoking and excessive alcohol consumption can exacerbate both conditions by increasing oxidative stress and inflammation [5,207,243]. Hence, the analysis of metformin’s effect on neurodegenerative diseases is a subject of considerable current interest.

In a Veterans Administration cohort study comparing different diabetes medications, metformin monotherapy was associated with lower dementia risk compared to sulfonylureas [244]. On the other hand, a meta-analysis from 2018 found that metformin use was associated with a reduced risk of dementia compared to no pharmacological treatment, with longer treatment duration showing greater benefit [222]. Similarly, in trials of patients with mild cognitive impairment and early dementia, metformin demonstrated potential cognitive benefits, including improved executive functioning and trends toward improved learning/memory [236]. Recent Alzheimer’s Disease Neuroimaging Initiative (ADNI) data have shown that diabetic patients treated with metformin during early AD stages exhibited better cognitive performance and cerebrospinal fluid biomarker profiles, with treatment associated with higher amyloid beta and lower tau/phosphorylated tau levels [245]. However, some studies have found no protective effect or even suggested increased risk—a meta-analysis examining metformin use and AD risk found no clear overall association (odds ratio (OR) 1.17, 95% confidence interval (CI) 0.88–1.56), though subgroup analysis revealed increased AD risk among Asian populations [246]. The heterogeneous treatment effects may be explained by factors such as timing of intervention, presence of neuropsychiatric disorders, and concurrent medication use, highlighting the need for careful patient selection when considering metformin for cognitive protection [247]. Building on this mixed evidence, the ongoing MET-FINGER randomized controlled trial is evaluating a novel precision prevention approach combining lifestyle intervention with metformin in non-diabetic older adults at increased dementia risk, which may provide crucial evidence for implementing innovative combination strategies for AD prevention [248]. Taking into account the research published in ClinicalTrials.gov, there are two completed studies on this topic, one focusing on AD biomarkers and the second on the topic of cognitive impairment [249,250]. There are two other studies, one assessing the use of metformin in AD prevention (active, not recruiting), and the second being a cross-sectional pilot study of brain imaging biomarkers in the Diabetes Prevention Program (terminated) [9,251].

Current research directions intend to clarify contradictory findings by investigating distinct biological targets of metformin in the brain, the balance of positive and negative effects of this drug, as well as possible genetic variables that may alter individual reactions to metformin therapy [252].

### 5.3. Parkinson’s Disease

Metformin offers neuroprotection in Parkinson’s disease through several pathways. It primarily activates AMPK, a key energy sensor, boosting mitochondrial biogenesis (via PGC-1α), improving energy metabolism, and reducing oxidative stress in dopaminergic neurons. Metformin also has anti-inflammatory effects, suppressing microglial activation and reducing pro-inflammatory cytokines (TNF-α, IL-1β, IL-6) [4,6]. It enhances autophagy (via mTOR inhibition and AMPK activation), clearing damaged components and protein aggregates. Furthermore, metformin modulates genes involved in cellular stress response (Nrf2), boosting antioxidant defenses [253,254,255].

Clinical evidence for metformin’s efficacy in Parkinson’s comes from observational studies and trials. Epidemiological studies suggest a reduced Parkinson’s incidence in diabetic metformin users [256,257]. A retrospective study found that metformin users have lower risk of developing Parkinson’s. Interventional trials show promising results in motor function improvement (based on the Unified Parkinson’s Disease Rating Scale), though evidence is somewhat mixed. Ongoing phase II trials are exploring metformin’s potential as a disease-modifying therapy, especially in early-stage Parkinson’s [11,258]. Clinicaltrials.gov also provides a study which analyzes the correlation between idiopathic Parkinson’s disease and diabetes mellitus in Egyptian elderly patients; however, its status is unknown [259].

Metformin’s impact on α-synuclein is crucial to its potential therapeutic benefits. It influences α-synuclein aggregation and clearance through multiple mechanisms. It can directly interact with α-synuclein monomers, potentially altering their conformation and reducing toxic oligomer formation [260,261]. Via AMPK activation, metformin enhances autophagic α-synuclein clearance (macroautophagy and chaperone-mediated autophagy). It also reduces α-synuclein phosphorylation at Ser129 (associated with aggregation and toxicity) and modulates α-synuclein expression (potentially epigenetically via AMPK). Metformin’s reduction of oxidative stress and inflammation indirectly affects α-synuclein aggregation [262,263,264].

Proteomic analyses show that metformin alters the α-synuclein interactome, potentially affecting its processing and degradation. Transgenic models show reduced α-synuclein aggregates and improved neuronal survival in metformin-treated animals. Its effect on α-synuclein propagation is under investigation, with preliminary evidence suggesting that it might affect pathogenic α-synuclein release and uptake. These findings support metformin’s potential to modify α-synuclein pathology in Parkinson’s disease [265,266].

These mechanisms have therapeutic implications, especially for metformin timing and dosing. Its multiple effects on α-synuclein suggest that early intervention might be most beneficial, as demonstrated in multiple preclinical studies [7,267]. In a rotenone-induced mouse model of Parkinson’s disease, metformin administration (300 mg/kg/day) attenuated the loss of tyrosine hydroxylase (TH+) neurons in the substantia nigra (SN), decreased cleaved caspase-3 and α-synuclein accumulation in the SN, and reduced the levels of malondialdehyde (MDA) and 4-hydroxynonenal (4-HNE), indicating decreased lipid peroxidation, compared to the rotenone-only group [268]. In *C. elegans* models of Parkinson’s disease, metformin treatment (10 mM) significantly decreased 6-OHDA-induced dopaminergic neurodegeneration, inhibited α-synuclein aggregation in transgenic worms expressing human α-synuclein, and upregulated expression of the antioxidant gene sod-3. Specifically, 10 mM metformin reduced α-synuclein aggregation and improved lipid deposition in the NL5901 strain expressing α-synuclein yellow fluorescent (YFP) fusion proteins [269].

### 5.4. Huntington’s Disease

Metformin has emerged as a promising therapeutic agent for Huntington’s disease (HD) through multiple mechanisms of action, especially its activation of AMP-activated protein kinase [270]. Metformin inhibits the MID1/PP2A/mTOR protein complex, reducing mutant huntingtin (mHtt) mRNA translation and reducing toxic protein levels [271]. It activates AMPK, improving cognitive function and reducing neuropsychiatric symptoms in HD models [272]. Metformin also influences autophagy and mitochondrial dynamics, mitigating toxic effects from mHtt aggregation. It enhances brain-derived neurotrophic factor levels and reduces inflammation-related glial activation [273,274].

In the Hdh150 mouse model, metformin administration during very far from disease onset (VFDO) stages reversed early cortical network dysfunction, including aberrant neuronal hyperactivity and enhanced synchronicity, while also normalizing anxiety-related behavioral changes [271]. These findings collectively indicate that metformin may have therapeutic potential for HD, particularly when administered early in the disease course, before substantial neuronal damage occurs. Preclinical studies have demonstrated that metformin reduces mHTT aggregation, prevents neuronal death, and improves neuropsychiatric phenotypes in HD models. In the zQ175 mouse model, metformin treatment reduced nuclear mHTT aggregates in the striatum, partially restored brain-derived neurotrophic factor (BDNF) levels, and attenuated glial activation [275]. These neuroprotective effects appear to be mediated through AMPK-dependent mechanisms and enhanced autophagy, as demonstrated in *C. elegans* models where metformin reduced polyQ aggregation and improved neuronal function in an AMPK- and lysosome-dependent manner. However, the timing of AMPK activation appears critical, as late-stage activation may have detrimental effects. A post hoc analysis of the Enroll-HD database revealed that HD patients taking metformin showed better cognitive performance compared to non-metformin users, particularly in verbal fluency and Stroop interference tests [276]. These findings have led to an ongoing phase III clinical trial (which can be found on Clinicaltrials.gov) evaluating metformin’s efficacy in treating cognitive decline in HD patients, with results that were expected in 2024; however, its status is currently unknown [10].

Metformin is being investigated not only as a treatment for existing symptoms but also as a preventive measure for those at risk of developing Huntington’s disease. The existing body of research supports its potential as a promising therapeutic option, particularly due to its established safety profile from long-term use in diabetes management.

### 5.5. Multiple Sclerosis

Multiple sclerosis treatment strategies continue to evolve, with increasing interest in repurposing established medications. The following section concentrates on the potential therapeutic applications of metformin in MS treatment, with emphasis on its molecular mechanisms, immunomodulatory effects, and clinical implications. Current evidence suggests that metformin’s activation of AMPK and modulation of the mTOR pathway may provide beneficial effects in MS through multiple mechanisms, including immunomodulation, neuroprotection, and metabolic regulation [277,278,279,280].

Research evidence from randomized controlled trial demonstrates metformin’s multifaceted therapeutic potential in multiple sclerosis through several key mechanisms, as reported by Keersmaecker et al. [281]. The drug exhibited significant remyelinating properties via the enhancement of oligodendrocyte precursor cell function and differentiation, mediated through AMPK pathway activation. The study has established metformin’s ability to rejuvenate aged oligodendrocyte precursor cells (OPCs) and restore their proliferative capacity, particularly in white matter regions. Metformin demonstrated considerable immunomodulatory effects, evidenced by reduction in inflammatory markers and T2 lesion formation. The drug’s neuroprotective capabilities were attributed to its antioxidant properties, mitochondrial function optimization, and downregulation of pro-inflammatory microglial activity through Mac-3 mRNA modulation [281]. Clinical observations support these mechanisms, with studies reporting reduced disability progression in MS patients receiving metformin, particularly notable in cohorts with comorbid type 2 diabetes. A pilot study (N = 50) demonstrated significant reduction in new T2 hyperintense lesions alongside favorable immunological changes. Although only a small percentage of metformin crosses the blood–brain barrier, this amount appears to be sufficient to potentially exert a beneficial effect in the treatment of multiple sclerosis, as suggested by similar results obtained by Gilbert and Livingstone et al. Their experimental study demonstrated that metformin administration at disease onset significantly reduced neurological impairment and protected myelin integrity in experimental autoimmune encephalomyelitis through microglial modulation, without affecting oligodendrocyte precursor dynamics. However, delayed treatment showed limited efficacy, suggesting metformin’s potential therapeutic value in multiple sclerosis may depend on early intervention. The drug’s established safety profile and demonstrated neuroprotective properties warrant further investigation for clinical translation [282].

While current disease-modifying therapies (DMTs) have shown significant efficacy, there remains a need for treatments that can address both the inflammatory and neurodegenerative aspects of MS [283,284]. Metformin has emerged as a promising candidate for MS treatment due to its diverse mechanisms of action and well-established safety profile. Metformin’s primary mechanism of action involves the activation of AMP-activated protein kinase, a fundamental regulator of cellular energy homeostasis [281,284]. In the context of MS pathophysiology, AMPK activation produces several beneficial effects. The pathway reduces inflammatory cytokine production while enhancing mitochondrial function and glucose metabolism in neural cells [285,286]. Additionally, AMPK activation modulates T-cell responses, potentially affecting the autoimmune component of MS [287,288]. The mammalian target of rapamycin pathway represents another crucial mechanism through which metformin may exert therapeutic effects in MS [289,290]. Metformin’s inhibition of mTOR leads to decreased inflammatory cell proliferation and regulated immune cell differentiation, as was shown in a recent study on *Hiradentis suppurtiva*, using the same mTOR pathway [216]. Furthermore, this modulation impacts myelin regeneration processes and influences neuronal survival mechanisms, both of which are critical in MS pathophysiology [290]. Metformin demonstrates significant effects on T-cell function, including the suppression of pro-inflammatory T-cell responses and promotion of regulatory T-cell development [291]. The drug modulates the Th17/Treg balance, which is crucial in autoimmune conditions [280]. The effects of metformin on B-cells include modulation of activation and differentiation processes, reduction in antibody production, and influence on B-cell-mediated inflammation. These actions may contribute to reducing the adaptive immune response involved in MS pathogenesis [292]. Metformin significantly impacts microglial cell function by reducing activation and decreasing pro-inflammatory cytokine production. It promotes an anti-inflammatory phenotype and enhances neuroprotective functions, potentially contributing to reduced neuroinflammation in MS [293,294]. The drug demonstrates positive effects on blood–brain barrier integrity, reducing immune cell infiltration and modulating endothelial cell function [44,295]. Current research suggests promising therapeutic potential for metformin in multiple sclerosis. Provided studies indicate that metformin’s effects may help limit central nervous system inflammation in MS. The drug’s metabolic effects, including improved glucose utilization, enhanced insulin sensitivity, and reduced oxidative stress, target key pathogenic mechanisms in MS. While early clinical observations suggest potential benefits in reducing relapse frequency and slowing disability progression, these findings primarily come from basic research [296].

Currently there are seven trials available at Clinicaltrials.gov. These studies explore metformin’s ability to promote remyelination, modulate immune responses, and impact neurodegeneration in various MS subtypes, with trials ranging from Phase I to Phase IIa and encompassing both pediatric and adult populations. Trial statuses include “recruiting”, “completed”, “unknown status”, and “not yet recruiting”, reflecting the ongoing and evolving nature of this research [297,298,299,300,301,302,303]. Larger clinical trials are needed to definitively establish metformin’s therapeutic value, particularly its potential to enhance existing MS treatments and provide neuroprotective effects. The metabolic benefits may be especially relevant for MS patients, who often show altered metabolic profiles, but human studies are required to confirm these promising preclinical results.

### 5.6. Metformin vs. Other Treatments

Metformin presents a multifaceted therapeutic approach for neurodegenerative disorders and multiple sclerosis, exhibiting distinct advantages over conventional treatments. In neurodegenerative diseases, while other insulin sensitizers like pioglitazone improve insulin sensitivity, metformin’s broader impact on oxidative stress, mitochondrial dysfunction, and protein aggregation, through AMPK activation and mTOR inhibition, offers a more comprehensive neuroprotective strategy [12,304]. Compared to antioxidants like vitamin E, which primarily target oxidative stress, metformin additionally modulates cellular aging and inflammatory pathways, potentially offering disease-modifying benefits beyond symptom management [305,306,307]. In MS, metformin’s capacity to promote remyelination and reduce neuroinflammation, through mechanisms targeting mTOR and Nrf2, distinguishes it from simple antioxidant therapies like N-acetylcysteine. While disease-modifying drugs like interferon-beta remain the cornerstone of MS treatment, metformin’s potential as an adjunct therapy, though still under investigation in trials like MACSiMiSE-BRAIN, suggests a valuable role in addressing metabolic comorbidities and potentially enhancing remyelination [281,282]. However, variability in clinical responses and the need for larger, well-controlled trials emphasize the importance of further research to fully elucidate metformin’s therapeutic potential in these complex neurological conditions.

### 5.7. Biomarkers for Predicting Metformin Response

#### 5.7.1. Genetic Biomarkers

Polymorphisms in genes encoding transporters responsible for metformin pharmacokinetics have shown consistent associations with treatment response. The *SLC22A1* gene, encoding the organic cation transporter 1 (OCT1), contains several reduced-function variants (R61C, G401S, M420del) that correlate with decreased metformin efficacy. In the Rotterdam study, carriers of two reduced-function SLC22A1 alleles demonstrated significantly smaller HbA1c reductions compared to carriers of functional alleles [308]. Similarly, variants in the *SLC22A2* (OCT2) and *SLC47A1/2* (MATE1/2) genes affect metformin excretion and plasma concentration. The common rs316019 variant in *SLC22A2* has been linked to reduced metformin clearance, while the rs2289669 G>A polymorphism in *SLC47A1* correlates with enhanced metformin efficacy, particularly in Asian populations [309].

Perhaps most significantly, a genome-wide association study identified the rs11212617 variant near the *ATM* gene as strongly associated with metformin response. This finding, replicated across multiple cohorts, suggests that carriers of the C allele achieve better glycemic control with metformin treatment. Mechanistic studies indicate *ATM* may influence AMPK signaling, a central pathway in metformin’s action [310].

#### 5.7.2. Metabolic Biomarkers

Baseline glycemic parameters consistently predict treatment outcomes, with higher pre-treatment fasting glucose and HbA1c values associated with greater absolute reductions following metformin therapy. The trial demonstrated that baseline insulin sensitivity and beta-cell function measurements significantly predicted long-term glycemic response [311]. Metabolomic approaches have identified specific plasma markers with predictive potential. Elevated baseline levels of branched-chain amino acids (particularly valine, leucine, and isoleucine) and acylcarnitines correlate with suboptimal metformin response. Alpha-hydroxybutyrate, an early marker of insulin resistance, shows promise as a predictor of efficacy [312].

#### 5.7.3. Clinical Biomarkers

Several readily accessible clinical parameters demonstrate associations with metformin response. Lower BMI consistently predicts better treatment outcomes [313]. Visceral adiposity, as measured by waist circumference or imaging techniques, may offer superior predictive value compared to BMI alone, potentially explaining observed gender differences in response [314]. Age and diabetes duration significantly influence treatment efficacy. Younger patients and those with shorter diabetes duration prior to metformin initiation typically achieve greater HbA1c reductions [315]. The Diabetes Prevention Program clearly demonstrated superior efficacy of metformin in preventing diabetes progression among younger individuals with higher BMI [316].

## 6. Research Gaps, Emerging Technologies, and Future Perspectives

Significant research gaps remain in understanding the complex pathophysiology of neurodegenerative diseases and optimizing therapeutic interventions, particularly regarding metformin’s potential preventive role [317]. A critical challenge lies in identifying patients at risk of developing neurodegenerative conditions or those in early disease stages, when therapeutic interventions like metformin might be most effective [236,237]. This is especially relevant given that type 2 diabetes mellitus (T2DM), a primary indication for metformin, is preceded by long-term insulin resistance, hyperglycemia, and oxidative stress—all key factors predisposing to neurodegenerative diseases [5,207,243]. Currently, we may be missing opportunities for metformin’s preventive benefits due to delayed implementation and incomplete understanding of the temporal relationship between metabolic dysfunction and brain pathology [200,201].

The complex interplay between protein aggregation, neuroinflammation, mitochondrial dysfunction, insulin resistance, and metabolic disruption makes it difficult to pinpoint primary pathogenic mechanisms and optimal intervention timing [318]. This is particularly relevant for metformin therapy, as its mechanisms of action intersect with multiple pathological pathways [221,222]. Translating preclinical findings to effective treatments remains challenging due to limitations in current animal models and high clinical trial failure rates. The lack of reliable early biomarkers and incomplete understanding of cell type-specific vulnerabilities hinder both research and clinical practice, potentially delaying the implementation of preventive strategies with metformin and other therapeutics [317].

Novel technologies are revolutionizing the field with potential to address these gaps. Single-cell RNA sequencing, spatial transcriptomics, and advanced proteomics offer unprecedented insights into cell type-specific responses and disease mechanisms, which could help identify early molecular signatures indicating the need for preventive metformin therapy [130,319]. Artificial intelligence and machine learning facilitate complex data analysis, drug response prediction, and multi-omics data integration, potentially enabling better patient stratification for metformin treatment [320]. These technologies could help identify which patients might benefit most from early metformin intervention, even before clinical manifestations of neurodegeneration.

Human brain organoids, microfluidic organ-on-chip platforms, and CRISPR-based genome editing are advancing disease modeling and therapeutic development, allowing better understanding of metformin’s effects on neural tissue [130,319,320]. Advanced imaging techniques are improving disease monitoring and could help track early pathological changes when metformin intervention might be most beneficial. These technologies are also being leveraged to enhance drug delivery across the blood–brain barrier, particularly relevant for medications like metformin where optimizing BBB penetration remains a crucial challenge [71,72,73].

The field is moving toward precision medicine based on genetic and biomarker profiles, which could help identify patients who might particularly benefit from metformin’s neuroprotective effects [321,322,323,324]. This individualized approach is essential given that metformin’s effectiveness may vary among different patient populations and disease stages. A critical challenge remains the timing of therapeutic intervention, as evidence suggests that metformin may be most effective in early disease stages or as a preventive measure, particularly in patients with metabolic dysfunction who are at higher risk for neurodegeneration [325,326,327].

Key research priorities should include:

- Developing early detection biomarkers specifically focused on identifying patients who might benefit from preventive metformin therapy [236,237],
- Understanding the temporal relationship between metabolic dysfunction and neurodegeneration to optimize metformin intervention timing [207],
- Investigating the link between systemic inflammation, insulin resistance, and neurodegeneration to better target metformin’s therapeutic effects [200,201],
- Establishing clear criteria for preventive metformin use in patients with metabolic dysfunction at risk for neurodegeneration [241,243],
- Developing more sensitive methods to monitor brain health in T2DM patients to guide metformin therapy optimization [324,325],
- Gene therapy advancements for neurodegenerative conditions or combined with stem cells [114],
- Personalized medicine approaches integrating these novel technologies with established treatments like metformin [114].

These advances require robust collaboration between clinicians, researchers, and industry partners, along with significant research investment. The focus should be on translating basic research findings into practical clinical applications, particularly regarding the preventive use of metformin in at-risk populations [324,325,326]. This approach could maximize the therapeutic potential of this well-established medication while developing new strategies for early intervention in neurodegenerative diseases.

## 7. Conclusions

Based on the extensive literature review, metformin shows promising potential in treating various neurodegenerative conditions through multiple mechanisms. Research demonstrates its ability to activate AMPK pathways, enhance antioxidant defenses, and provide anti-inflammatory effects as well as increase brain sensitivity to insulin [4,12].

The current clinical evidence base for metformin in neurodegenerative diseases is predominantly comprised of observational studies, which suggest a reduced risk of neurodegeneration in metformin-treated patients, though some findings remain mixed [7,8,246]. While these observational data are encouraging, there is a notable scarcity of interventional clinical trials. For Huntington’s disease and multiple sclerosis, current evidence largely relies on basic research and animal studies, highlighting the need for more robust clinical investigations.

The drug’s neuroprotective effects are particularly notable in Parkinson’s disease, where it influences α-synuclein aggregation and clearance, and in multiple sclerosis, where it modulates neuroinflammation and immune responses [260,261,277,278]. In Huntington’s disease, metformin shows promise through its effects on mutant huntingtin protein levels and mitochondrial function [270,271].

However, significant research gaps remain, particularly in understanding the optimal timing of intervention and patient-specific responses [317]. Emerging technologies, including single-cell sequencing and advanced imaging, are providing new insights into disease mechanisms and potential therapeutic approaches [319,320]. Moving forward, the field is trending toward precision medicine approaches and multi-modal therapies [325,326].

Notably, metformin holds several distinct advantages over newer therapeutic agents. As a long-established medication, it has well-characterized pharmacodynamic and pharmacokinetic properties, along with a thoroughly documented safety profile. Its demonstrated neuroprotective effects, combined with its low cost and extensive clinical experience, make it an attractive candidate for broader investigation. These factors strongly support the expansion of clinical trials examining metformin’s preventive and therapeutic potential in neurodegenerative diseases, particularly as part of comprehensive treatment strategies.

It is important to acknowledge that several studies have reported no significant benefits or even adverse effects of metformin in neurodegenerative contexts [328,329]. These contradictory findings may be attributed to various factors, including heterogeneity in study populations, differences in disease stages, varying comorbidity profiles, and inconsistent dosing protocols [222,330].

Methodological limitations in existing studies further complicate the interpretation of these mixed results. Many observational studies face challenges with confounding variables, inadequate control for diabetes severity, and selection bias that may mask or artificially enhance metformin’s apparent effects [56,331]. Additionally, genetic polymorphisms affecting metformin transporters and receptors may significantly influence the treatment response, potentially explaining inconsistent outcomes across different populations [332].

The drug’s relatively limited blood–brain barrier penetration presents another potential explanation for null findings, as insufficient central nervous system concentrations may fail to achieve therapeutic effects in some patients, particularly those with compromised blood–brain barrier integrity [169]. Future studies should address these limitations through more rigorous methodologies, careful patient stratification, standardized dosing regimens, and longer follow-up periods to better characterize metformin’s true potential in neurodegenerative disease management [224].

## Figures and Tables

**Table 1:** Description of key mechanisms of metformin. Metformin enhances glucose metabolism through multiple mechanisms, including suppression of hepatic glucose production, increased peripheral insulin sensitivity via GLUT4 translocation, and modulation of intestinal glucose transport and gut microbiome. At the cellular level, it regulates energy metabolism by altering NADH/NAD+ ratios and activating AMPK pathways, while affecting mTOR signaling. It also provides anti-inflammatory benefits through reduction of pro-inflammatory cytokines and oxidative stress.

Key Mechanism	Description of Function
Hepatic Glucose Production	Suppression of gluconeogenic gene expression, including *PEPCK*, *GPD2*, with the latter affecting cellular NADH/NAD+ ratio [25,31,85], At supratherapeutic concentrations only: Modulation of mitochondrial function through inhibition of complex I of the electron transport chain [23,86], AMPK-dependent reduction in hepatic lipogenesis and enhancement of fatty acid oxidation [87], Protection against glucotoxicity and lipotoxicity-induced beta cell apoptosis [88,89].
Peripheral Insulin Sensitivity	Upregulation of GLUT4 translocation to plasma membranes [90], Enhancement of IRS signaling [91], Modification of membrane fluidity affecting insulin receptor function [92,93], Improvement of glucose-stimulated insulin secretion under chronic hyperglycemic conditions [94].
Intestinal Glucose Metabolism	Alteration of the gut microbiome composition [95], Modulation of enterocyte glucose transport [96], Enhancement of GLP-1 secretion [97].
Cellular Energy Metabolism	Reduction in cellular NADH/NAD+ ratio through inhibition of mitochondrial glycerol-3-phosphate dehydrogenase [28], Activation of AMPK through increased AMP/ATP ratio [98], Downregulation of mTOR signaling pathways [99,100].
Anti-inflammatory Effects	Suppression of microglial activation and reduction of pro-inflammatory cytokines (TNF-α, IL-1β, IL-6) [4], Modulation of inflammatory signaling pathways [101], Reduction of oxidative stress-induced inflammation [102], Preservation of beta cell mass and function through reduction of oxidative stress [103].

Abbreviations: phosphoenolpyruvate carboxykinase (PEPCK), glucose-6-phosphatase, and glycerol-3-phosphate dehydrogenase 2 (GPD2), glucose transporter type 4 (GLUT4), insulin receptor substrate (IRS), glucagon-like peptide-1 (GLP-1), mammalian target of rapamycin (mTOR), tumor necrosis factor alpha (TNF-α), interleukin 1 beta (IL-1β), interleukin 6 (IL-6).

## Data Availability

Data sharing is not applicable, because no datasets were generated or analyzed during the review article.
